# Send It In

**Published:** 1917-01

**Authors:** 


MRS. F. E. DANIEL, Publisher and Managing Editor.

### Send It In.

"If you have a bit of news,
Send it in.
Or a joke that will amuse,
Send it in.

A story that is true,
An incident that's new,
We want to hear from you!
Send it in.

Will your story make us laugh?
Send it in.
Send along a photograph,
Send it in.

Never mind about your style
If the story's worth the while,
And may *help* or cause a smile,
Send it in."

—*Clipped.*

